# Level of interest among Belgian consumers of the cultural, environmental, ethical, and global benefits of sustainable beekeeping management

**DOI:** 10.1016/j.heliyon.2024.e40928

**Published:** 2024-12-09

**Authors:** Jatziri Mota-Gutierrez, Stefano Massaglia, Valentina Maria Merlino, Federica Rosa, Andrea Viberti, Simone Blanc

**Affiliations:** a Department of Veterinary Sciences, University of Turin, Largo Paolo Braccini 2, 10095, Grugliasco, Turin, Italy; b Department of Agricultural, Forest, and Food Sciences, University of Turin, Largo Paolo Braccini 2, 10095, Grugliasco, Turin, Italy; c RV STUDIO S.R.L, Via Montello 2/B, 12042, BRA, CN, Italy

**Keywords:** Honeybee management, Consumer awareness, Sustainable apiculture, GLMM

## Abstract

This study aimed to explore the consumers’ level of interest in environmental, ethical, cultural, and global claims associated with sustainable beekeeping management and identify which factors influence perceptions of sustainable management and honey purchase. 1100 Belgian respondents were surveyed on their honey purchasing behavior and interest in the benefits of sustainable beekeeping management, complemented with socio-demographic questions. The data were evaluated using descriptive, non-parametric and multivariate statistics. The findings indicate that age and honey purchasing habits had a significant effect on the level of interest in the benefits of sustainable beekeeping management. Participants in the late middle-aged group who buy honey displayed greater interest in all the mentioned benefits of sustainable beekeeping practices when compared to their younger counterparts. Early and late middle-aged participants demonstrated a heightened level of interest in environmental claims concerning the advantages of sustainable beekeeping management. This study provides critical insight into consumers’ perceptions towards sustainable apiculture and helps to identify environmentally sustainable claims to empower consumers to make conscious and informed choices and protect the environment.

## Highlights

• Beekeeping practices impact cultural, ethical, environmental and global aspects.
• Consumers responded positively to the benefits of sustainable beekeeping management.
• Most of our respondents purchase honey on average once *per* year or once *per* month.
• Consumers need a clear, brief, and regulated sustainability honey label.
• Poor management of beekeeping practices is considered an external stressor to bees.

## Graphical abstract

**Figure:**
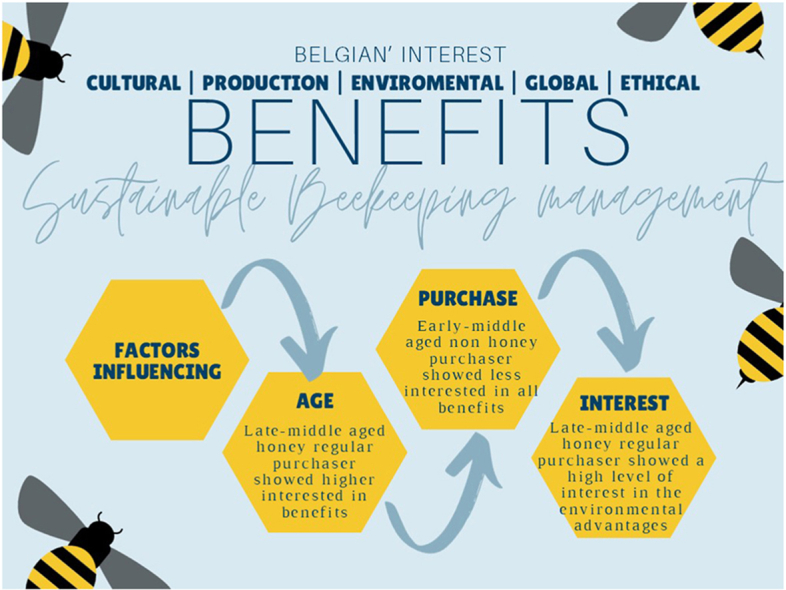

## 1 Introduction

A major contributor to the health of the environment is bees. Beekeeping provides human and animal nutrition (honey, bee pollen, propolis), medicine (propolis, bee venom), materials (beewax), and can contribute to several agroecosystem services such as regulating pollination, pest and disease control, climate regulation. From the human perspective, bees contribute to food security, medical resources and they support farmer and beekeeper livelihoods, contributing to social and cultural values [1].Despite the great benefits offered by bees, humans often harm bee health, consequently affecting the quality and quantity of bee products and several agroecosystem services such as pollination, pest and disease control, and climate regulation [[1], [2], [3]]. The main external stressors to bees include land-use changes, disease and pest, arbitrary use of chemicals, climate change, increased monocultures, the introduction of invasive pathogens, and poor management practices [4]. Several initiatives have been implemented to boost beekeeping activities using a system-based approach to bring together different stakeholders to enhance research and development of beekeeping technologies, compiling guidelines to promote proper, efficient, and sustainable apiculture management, and raising awareness of bee health [2,[5], [6], [7], [8]].

The Good Beekeeping Practices guidelines for sustainable apiculture emerged in response to the need to promote the sustainability of pollination, apitherapy and apitourism services, honey, wax, propolis and pollen production, they also support the livelihoods of small-scale producers and helps to maintain a healthy environment and achieve Sustainable Development Goals (SDGs) across the world, focusing in rural areas [2]. Guidelines for best practices provide instructions on the implementation of eco-conscious beekeeping management, encompassing good hygiene and harvesting techniques, pollinator-friendly environmental enhancements, the adoption of integrated pest management, reduction in pesticide exposure, monitoring, and measures to curb disease transmission, all aimed at mitigating high beekeeping losses and the decline of wild bee populations [2,9,10]. According to Cass et al., 2022*,* beekeepers, farmers, and landowners believe that implementing sustainable pollinator management is important and enthusiastically support these practices, but knowledge of tools to implement them is lacking [11]. However, as far as the authors are aware, there is no published analysis of the consumers’ perception of the benefits of sustainable beekeeping management and the factors affecting this level of interest.

European beekeepers and stakeholders have noted that beekeeping management is evolving in response to the challenges posed by climate change, necessitating a more urgent adjustment in practices, especially along the North-South gradient in Europe [12]. Sustainable or organic beekeeping is based on complying the standards of quality and respect towards the environment. This practice is employed in nearly 954 000 beehives across Europe [13]. Research indicates a favorable trend in the demand for organic bee products and honey, which will serve as an incentive for beekeepers to gradually transition from conventional to sustainable beekeeping management, as suggested by Willer et al., 2021 [14]. When considering “sustainable beekeeping management” for the transformation of bee products, three main research questions emerge: what factors influence the honey consumer's purchasing decision, particularly in light of environmental concerns, political considerations, and economic factors in Europe? Do consumers show an interest in the benefits of sustainable beekeeping management? What factors influence consumers’ perceptions of the benefits of using sustainable beekeeping practices?

Existing literature indicates a growing interest of consumers in environmentally friendly, local, and organic food, especially in European countries and the United States [15,16]. From the consumers perspective, honey is perceived as healthy, safe, and environmentally friendly product. Additionally, honey is as a super food because of its nutritional value and health-promoting properties; however, this product can be found in different colors and flavors, which is determined by the type of nectar that bees collect from floral sources [17]. The color, origin and organic certification of honey has been indeed found to be a main driver for honey [18]. According to Sedik et al., Silver Generation consumers prefer dark color [19]. With regards to the type of honey, studies suggest that the most prefer honey among consumers are polyfloral and multifloral honey, acaia honey, lime honey, honeydew and mountain flowers [[19], [20], [21], [22]]. Moreover, it has been showed that liquid honey, propolis, royal jelly and bee pollen were the most preferred honey flavors among Slovak consumers [19]. Research suggest that individuals’ demographic factors affect preferences for raw, organic, or flavored honey variations [23,24]. In addition, consumers are increasingly drawn to honey due to its perceived health benefits, including antimicrobial properties and potential immune-boosting effects [25].

Several recent studies emphasize the significance of diverse purchasing factors influencing honey consumption. Studies have shown that prices, product quality, brand reputation impact the purchasing behavior [26]. Nascimento et al. (2022) explored the impact of packaging design on consumer choices, concluding that visually appealing and eco-friendly packaging significantly influences purchasing decisions [27]. Furthermore, the origin and authenticity of honey emerged as critical factors affecting consumer trust and willingness to pay premium prices [28]. According to Vapa- Tankosić et al. (2020) higher monthly household income positively influences the willing to pay (WTP) for organic honey, while on the other hand, the higher level of education has a positive influence on the WTP for local honey [29]. The evolving nutritional value of honey and its therapeutic benefits perception significantly influences consumer purchasing patterns and usage frequency [30], [25].

However, in the context of unfair trade practices in the market, honey-one of the best-known beekeeping products-is the third-most adulterated food products in the world [31]. Honey adulteration has negatively impact both consumers and honey producers. From the consumer perspective, adulterated honey has increased consumers trust and preference towards traceable honey [32,33]. A recent study indicates that half of all Romanians are aware of honey adulteration, elderly people in particular are more aware of honey adulteration and are aware of better indicators of honey quality [32]. Traceable honey is perceived as safer, as having a better taste and flavor and a higher quality than standard products [34]. From the honey producer’s perspective, the rising trend of hive-to-honey traceability has a positive impact on the short supply chain, increasing purchases from beekeepers situated at markets, fairs, or direct delivery [32].

While much attention has been placed on understanding consumers’ food preferences from a specific place of origin, a certain food production method, local brands, and organic or sustainable certifications, consumers find it hard to translate their environmental concerns into their purchasing behavior [35,36]. Research suggests that consumers lack information and knowledge on food-related sustainability topics [37]. Nonetheless, the effectiveness of enabling informed decisions requires first understanding the relevant knowledge gap. Providing relevant skills, knowledge, and decision tools to consumers will inevitably influence food demand [38]. However, the efficacy of empowering good decisions requires first understanding the relevant knowledge gap.

In this article, we investigate the consumer’s perspective on the level of interest in the benefits of applying sustainable beekeeping management to address food communication challenges. This study utilizes honey buying as an illustration and examines the present purchase frequency, as well as how the factors leading to the non-consumption of honey, may be linked to the socio-demographic characteristics of the participants. The aim of this research is to analyse the perceptions of consumers in a sample of 1,100 participants, representative of the Belgian population, divided into balanced groups, regarding their attitudes towards five advantages of employing sustainable beekeeping management. Additionally, this study seeks to identify which of these benefits holds greater interest from the consumers' perspective. The benefits were presented to consumers as having a food production, cultural, ethical, environmental, or global effect. The main socio-demographic (gender and age) effect between the frequency of honey purchase and the perception of the benefits of sustainable beekeeping management were examine using generalized linear mix models. In applying a mix-method approach to data analysis, this study seeks to uncover how consumers’ associations and perceptions are affected by participants’ socio-demographics and their purchasing behaviour. This research contributes to understanding the market dynamics of sustainable apiculture, emphasizing the importance of targeted environmental claims in influencing informed consumer decisions and highlights the role of demographic factors in ecological consumerism.

### 1.1 Theoretical background

The increasing demand for credibility factors, including eco-friendliness, organic practices, and animal welfare in the agri-food sector, is elevating awareness about the environmental ramifications throughout the entire supply chain [39]. Although there is a rising trend among consumers in expressing their willingness to pay a premium for eco-friendly products, as reported by [40], a recent review has underscored the fact that consumers have limited awareness of the definitions of environmentally sustainable attributes, they also lack an understanding of how these attributes are associated with various stages in the food supply chain, and there are issues with the effectiveness of communication between producers and consumers [39]. The likelihood is that consumers will choose a food product increase when the information on the packaging is comprehensive [41,42]. However, misleading green claims lead consumers to feel deceived, which most likely translates to a significant loss of business and discourages environmental responsibilities. This study aimed to identify the level of interest in different environmental impacts on the use of sustainable beekeeping practices, thus contributing to supporting the advancement of the Sustainable Development Goal (SDG) for responsible consumption and production, which is vital for ensuring the well-being of present and future generations by fostering awareness of sustainable honey production.

Sustainable Development Goals (SDGs) are 17 objectives that recognise *“that ending poverty and other deprivations must go hand-in-hand with strategies that improve health and education, reduce inequality, and spur economic growth – all while tackling climate change and working to preserve our oceans and forests*” [43]. Research on the level of SDGs awareness and acceptance among consumers shows positive attitudes towards SDGs; however, a lack of knowledge of SDGs was reported [44,45]. Concerning the responsible consumption and production goal, in general, food choices are motivated more by individual preferences, such as taste, rather than altruism and concern for the environment or workers’ compensation [46,47]. Research shows that the working conditions along the agri-food chain emerged as one of the five factors defining consumers’ perception of fairness; however, this factor was less important than environmental attributes [46]. Previous research has shown that consumer awareness and associated environmental behaviour are influenced by the individual degree of necessity to protect the environment and sociodemographic characteristics [48]. In this context, it has been observed that young people, including millennials and Generation Z, frequently participate in pro-environmental actions as a component of their sustainable conduct [49,50].

From the market perspective, it is important to grasp, to what degree consumers know and care about the relationships between the benefits of using environmentally sustainable practices and the different impacts in the food system. Sustainable food claims and labels are complex [51]. International regulatory authorities are engaged in regulating and sorting the environmental claims of products, especially for the food industry to ensure that consumers are empowered to make conscious informed choices and protect the environment [52]. Despite the great efforts made to promote effective communication of environmental claims, research on the level of awareness of sustainable farming practices and interest among consumers is sparse [53]. More importantly, to the best of the authors’ knowledge, the level of consumer interest in sustainable beekeeping management was not considered previously in the literature.

Nevertheless, the increased awareness and a focus on greener and more sustainable practices have led to an increased preference for sustainable honey [54]. Honey products demand and consumption are dependent on product characteristics, the circumstances of purchasing decisions, socio-demographics, economics, knowledge, origin, and labels [[55], [56], [57], [58], [59]]. The most important criteria for purchasing honey are price, type of honey, quality and environmental aspects and health benefits [57,60,61]. According to Wojciechowska-Solis and Barska (2021) honey was considered a preferred sustainable product by Polish consumers [54]. One motivating factor that leads consumers to choose honey is their concern for the environment and animal welfare. They are also attracted to honey due to its lack of harmful substances in food production, as well as its perceived health and sustainability benefits [54,60]. The overall low awareness of production methods and the environmental impacts suggests that increasing the level of knowledge among consumers by providing concise and effective information is a valid strategy to bridge a knowledge gap. Therefore, it is essential to grasp the degree of consumer interest in their perceptions of sustainable honey products and the primary factors influencing honey purchases [54].

The present study makes a unique contribution to the literature to investigate the consumer perspective on the importance of the benefits of sustainable beekeeping practices. This study focusses on Belgium consumers as this country is a small honey consumer but is the second honey importer in EU. Consumers from Belgium worth to be investigated to understand the reasons why Belgian consumers do not consume honey and how these consumers perceived the importance of the benefits of sustainable honey beekeeping management as strategy to tailor product offering accordingly and create a more appealing brand, more importantly it could provide policymakers, and producers in EU understand how consumers process information and make decisions. This study seeks to uncover how consumers’ perceptions and frequency of honey purchase are affected by personal characteristics.

In this study, tested whether consumers’ perceptions towards sustainable beekeeping management would vary, with potentially more positive attitudes towards women and young individuals (H1). However, this impact may be evident in specific combinations of honey purchases and sustainable benefits, which will be revealed in the results as an interaction effect. We anticipate that the women and younger demographic will express more favorable views regarding the advantages of adopting sustainable apiculture and will exhibit a higher frequency of honey purchases compared to men and adult consumers. In addition, in this study, we expect that consumers’ perceptions towards sustainable beekeeping management would vary, with a higher level of interest in consumers buying honey (H2). In this context, consumers buying honey will report more positive attitudes toward sustainable beekeeping management than consumers who do not buy honey. Nonetheless, we hypothesize that, despite recent signs of heightened consumer awareness of organic food products [54], the interest in the environmental benefits of sustainable apiculture may not necessarily have a universally positive impact. It might, in fact, lead consumers to lean towards other specific claims. Therefore, our study tested whether the level of interest of the benefits of sustainable beekeeping management would vary depending on the participants’ purchase of honey (H3). In addition, we tested the level of interest of target environmental claims would vary according to age and purchase patterns.

## 2 Methods

### 2.1 Survey instrument

A total of 1160 surveys were completed (94.82 % response rate). We excluded 5.17 % (*n* = 60) of the sample due to missing data or not fulfilling quality checks (speeder flag). The final analysis included data from 1100 consumer. Average time of completing the survey was approximately 5–6 min. The online questionnaire was prepared using 14 questions and was divided into three sections, namely: 1) honey purchase habits (four questions); 2) sustainable beekeeping management perceptions (5 questions) and 3) socio-demographics (four questions), (Table S1). An initial pilot study was conducted with 50 respondents to identify problems relating to the wording of questions, errors and other problems experienced by respondents [62,63]. Minor modifications were made related to the phrasing of questions and response options. The revised questionnaire was then administered to three different people to evaluate the modifications and check whether further modifications were needed.

The survey began by asking participants about their honey-purchasing habits. The consumption of honey was assessed using a yes/no answer. If the response was not, the survey continued with a multi-choice question: Why you do not consume honey? A nominal multi-choice scale was measured to investigate the best-known attributes described by the general population for not consuming honey. The frequency of purchasing of honey was rated using a 7-point scale (1 = never to 7 = more than 5 times *per* week) to assess current purchasing honey habits. The place to buy honey was assessed using a nominal multiple-choice scale question described in Table 1.

**Table 1:** Measurement items.

Variable	Description
*S****ocioeconomic characteristics***	
Age	Consumer’s age
Gender	Consumer’s gender
Household size	Household members in count numbers
Purchase frequency	Interval scale (1 = never to 5 = more than 5 times a week
Place of purchase Honey	Large supermarkets
	Specialized stores
	Restaurants
	On-line
	None
Income	High
	Medium
	Low
*Perceived beekeeping benefits*	
How interest are you towards the following statements? Sustainable beekeeping managements … • promotes pollination, apitherapy and api-tourism. • produces honey, wax, propolis and pollen. • helps maintain a healthy environment. • supports the livelihoods of small-scale producers. • helps achieve the Sustainable Development Goals. Interval scale (1 = extremely uninterested to 10 = extremely interested)	

To test hypotheses, the survey then focuses on participants’ perceptions towards the interest in the benefits of sustainable honey beekeeping management using a 10-point interval scale (1 = not important at all to 10 = extremely important). Likert scales were used to verify the extend of consumers’ agreement with the questions described [64]. Positive cultural, ethical, environmental, and global perceptions were used to measure positive sustainable benefits using the five items according to FAO (2021) and described in Table 1.

As described in the introduction, food choices are dependent on socio-demographics. Thus, to explain the potential effect of socio-demographics on consumers’ perceptions and honey purchases, we surveyed participants’ age, gender, occupation, household composition, and annual household income, and completed a social and demographic description of the sample (Table 1). Participants were categorized by age into young (18–35 years), early middle-aged adults (36–55 years) and late middle-aged adults (older than 55 years) [65]. The annual income of participants was also divided into income groups broadly defined as low (less than 30 000 €), medium (between 30 001–45 0000 €), and high (more than 45 001 €) following the average annual wages in Belgium from 2001 to 2021 [66].

### 2.2 Data collection and management

Data were collected in June 2021 by an independent research team involved in the authorship. The Computer Assisted Web Interviewing (CAWI) was used as data collection methodology to select a balanced sample, representative of the Belgian market was urged. The study was conducted using a quota sample in which 1100 respondents in Belgium were surveyed. Quota sampling ensured that the sample reflected the Belgian adult population in terms of age and gender. The inclusion criterion was to person responsible for doing the groceries, individuals who agreed to participate and who gave their consent for data usage in the first question of the questionnaire and the individuals had to be over 18 years of age. Initially, the questionnaire was drawn up in English and was pre-tested by experts in consumer science. After their approval, the questionnaire was translated into French by native speakers to allow the respondents to fully understand the text and to enable maximum efficiency of the answers. The translated survey was pre-tested by a minimum of 5 subjects who were unrelated to the project to identify problems related to the phrasing of the questions, omissions, and other difficulties experienced by respondents, as shown previously [62,67]. Minor modifications were made related to the phrasing of the questions and response options. These pre-tests were used to obtain feedback for the researchers, and the questionnaire was adjusted accordingly. The online survey was anonymous, and the respondents electronically signed an informed consent form before participating in the survey and after having read a disclosure sheet that described the project and survey aims. The research was conducted in accordance with the guidelines defined in the Declaration of Helsinki, involving voluntary, informed participants aged 18 and above. The research was a personal initiative of the independent research team involved in the authorship. It was therefore not required an evaluation through the university ethics committee. All participants provided written informed consent to participate in the study and for the publication of their data in aggregated form.

### 2.3 Statistical analysis

Statistical analysis was performed using R software considering an alpha level of 5 % by an Applied Statistics Spec. A comparison of mean scores was used to assess associations between honey frequency purchase (interval variables, seven-point scale), level of interest of the benefits of sustainable beekeeping practices (interval variables, ten-point scale) and gender, age, and honey purchase. Statistical analyses were carried out using generalized linear mixed-effect models (*glmm*). Generalized linear mixed models (GLMMs) have been formulated to correct the assumptions made in Linear mixed models such as the straight relationship between some known function of the mean of *y* and the predictors *x* and random effects *z* (assumption check: plotting residual plots); constant variance (Levene’s test: *p*-value less than 0.05) and that random effects follow a normal distribution (Shapiro-Wilk test: P-value greater than 0.05). The assumptions that were met where 1) the observed *y* are independent, conditional on some predictors *x* (random sampling); 2) random effects *z* are independent of *y* (random sampling). Mixed models were chosen because of their ability to capture both fixed (Gender: women and men; Age: young, middle-aged adults, and late middle-aged adults; Purchase: Honey purchase and Avoidance; and Benefits: Cultural, Environmental, Global, Production, Ethical) and random effects (number of subjects, *n* = 1100). Power calculations for the sample size was used to ensure a significance level = 0.05 and f values = 0.4, using the “*pwr*” function (power = 1). F-critical values were calculated using the “*qf”* function. The *P*-values were adjusted using Bonferroni's method and when the mixed model revealed significant differences (*P* < 0.05), the least significant difference test was applied. Mixed models were built and evaluated according to Crawley using R [68]. Cronbach’s alpha was used to assess the internal consistency of the construct scales, where a value greater than 0.70 is usually recommended (Table 1). Correspondence Analysis (CA) was the multivariate test used for data analysis. CA is the most appropriate type of analysis for categorical data and is often used in the analysis of multiple answer frequency data (Greenacre, 1984; Olsen et al., 2015). CA was performed from the frequency table, obtained from the reasons indicated by consumers towards honey consumption, to visualize similarities and differences among the consumers using the *CA* function (*FactoMineR* package) and plotted through the *Factoextra* package of R version 3.3.2.

## 3 Results

### 3.1 Sample description

The resulting sample consisted of 1100 participants, representative of the Belgium population. The gender of our participants showed some heterogeneity. The social and demographic characteristics of the sample are described in Table 2.

**Table 2:** Sample demographic and personal information (*n* = 1100).

Characteristics	Respondents		Honey purchase	Honey avoidance
	n.	%	n.	n.
Gender				
Men	537	48.82	289	210
Women	563	51.28	262	258
**Age**				
Young	232	20.90	99	133
Early-middle aged	480	43.64	252	228
Late-middle aged	232	21.09	200	188
**Professions**				
Student	11	1.00	5	6
Unemployed	60	5.45	40	20
Employed	903	82.09	386	517
Retired	97	8.82	56	36
Business	29	2.64	19	11
**Annual income**				
Low	445	40.45	207	238
Medium	383	34.82	209	174
High	134	12.18	73	61
**Members**				
One	183	16.49	78	105
Two	398	35.86	206	192
Three	245	22.07	126	119
Four	192	17.30	95	97
More than 5	82	7.39	46	36
**Place of Purchase Honey**				
Large supermarkets	363	33.00		
Specialized stores	172	15.64		
Restaurant	24	2.18		
On-line	37	3.36		
None	504	45.82		
**Frequency of Purchase Honey**				
More than 5 times a week	26	2.36		
3–5 times *per* week	35	3.18		
1–3 times *per* week	77	7.00		
Once a month	161	14.64		
Once *per* year	417	37.91		
Special occasions	145	13.18		
Never	239	21.73		

### 3.2 Exploring avoidance of honey consumption

**Fig. 1:**
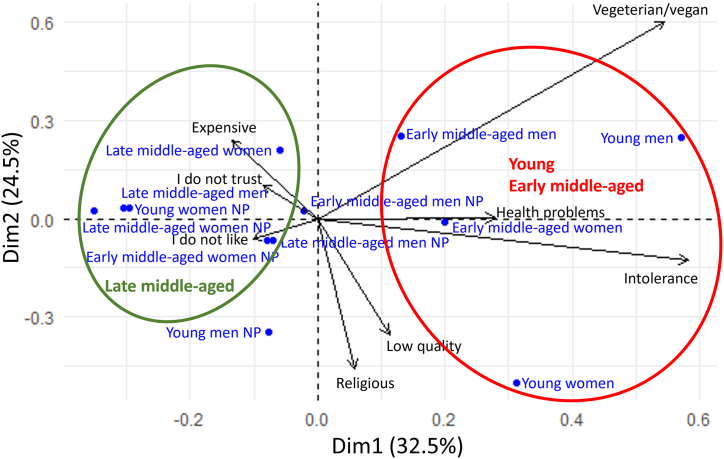
Age-related differences in the factors reported by consumers in relation to avoidance of honey consumption using corresponding analysis. Age groups are circled: red for young and early middle-aged and green for late middle-aged. NP: Non-purchasing.

The analysis performed with CA shown explains 32.5 % of the variance presented in the first axis and 24.5 % of the variance on the second. The analysis of similarities showed a significant difference in age (R = 0.542, *P* = 0.003) but not in gender (R = −0.151, *P* = 0.977, Fig. 1). A trend was observed where late-middle aged individuals tended to form associations linked to dislike, mistrust, and high expenses. Young and early middle-aged participants tended to be connected with perceptions related to intolerance and diet restrictions. While, health issues, religious factors, and the perception of lower product quality do not seem to be associated with age (Fig. 1). These results show clear differences in the free associations consumers link to avoidance of honey consumption. In addition, the attitudes of early and late middle-aged non honey consumers were strongly linked to their personal values and beliefs towards global issues related to food production.

### 3.3 Factors influencing the frequency of honey purchase

**Fig. 2:**
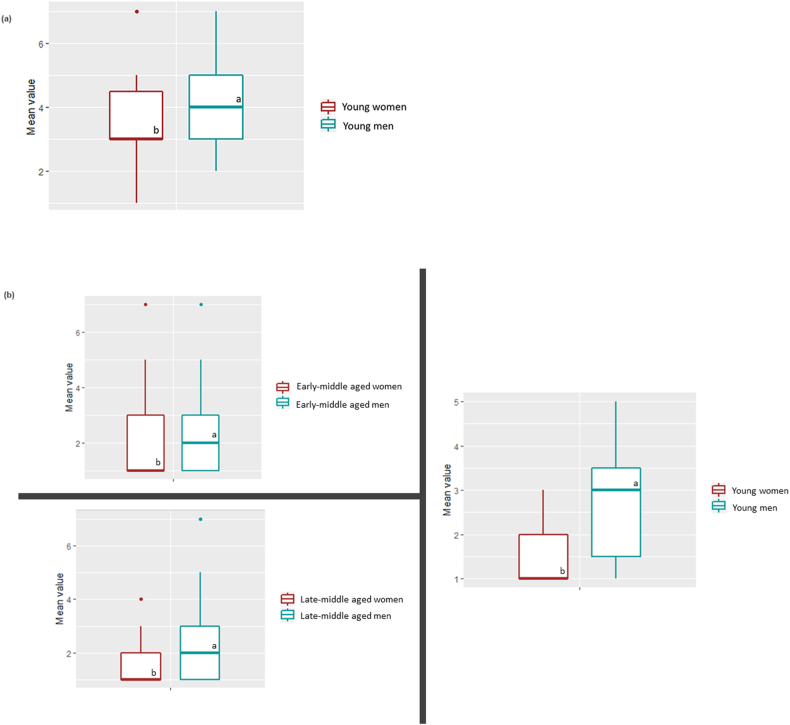
Gender effects on honey purchase frequency according toage and purchasing habits:**A)** Participants that purchase honey and **B)** Participants that avoid honey purchase. Different letters indicate statistical differences using least significant difference test (*P* < 0.05). *P*-values were adjusted using Bonferroni’s method.

The analysis of the frequency of honey purchase showed that 37.91 % of the respondents purchase honey on average once *per* year and 14.64 % of those surveyed do so once a month (Table 2). The analysis of the relationship between the frequency of honey purchase and the socio-demographic characteristics of the respondents showed that gender (F-statistic = 12.65, F statistic was greater than the F-critical value = 4.63, *P* = 0.0004) and age (F-statistic = 10.38, F statistic was greater than the F-critical value = 4.63, *P*= < 0.0001) significantly differentiated the frequency of honey purchase statistically. In this regard, men showed higher honey frequency purchase compared to women (3.10 and 2.71 respectively, score value interpretation 3 = once a year and 2 = occasionally), while young participants were also characterized high higher purchase patterns of honey higher than early and late-middle aged participants (3.18, 2.87 and 2.68 respectively, score value interpretation 3 = once a year and 2 = occasionally, P ≤ 0.05, Fig. 2). The differences in honey purchase frequency based on gender and age revealed that among individuals of the same age group, young, early-middle and late-middle aged men displayed a higher frequency of honey purchases compared to women (*P* ≤ 0.05, Table 3).

**Table 3:**
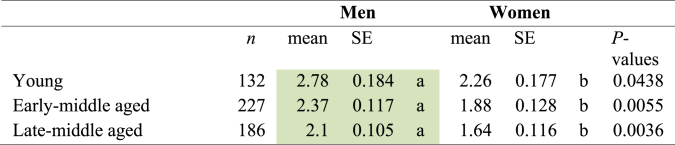
Gender effects on honey purchase according to age group.

5 = More than 5 times a week; 4 = 3–5 times a week; 3 = 1–3 times *per* week; 2 = once a month; 1 = once *per* year.

Abbreviation. **SE**= Standard error. Different letters indicate statistical differences related to gender differences using least significant difference test (P < 0.05). P-values were adjusted using Bonferroni’s method. Highlight in colour green are significantly higher mean values.

### 3.4 Factors influencing perceptions of the benefits of sustainable beekeeping management

In general, participants in the survey felt most of the information on sustainable beekeeping management listed in the survey ranged from very to quite interesting (Table 4). Age and honey purchase did have a significant effect on the perception of the benefits of sustainable beekeeping practices (*P* ≤ 0.05). Since the GLMM results for the composite perceptions towards all sustainable management items showed a significant age and purchase effect, data was disaggregated by age and honey purchase. Concerning the age effect, across all items, there was a significant difference in response between young and late-middle-aged participants who purchase honey and the ranking of their perceived benefits of sustainable beekeeping practices (*P* ≤ 0.05, Table 4A). Interestingly, the benefit obtained from using sustainable honey management to promote sustainable development goals did not lead to a significantly different attitude among participants who do not purchase honey, regardless of age (Table 4B).

**Table 4:**
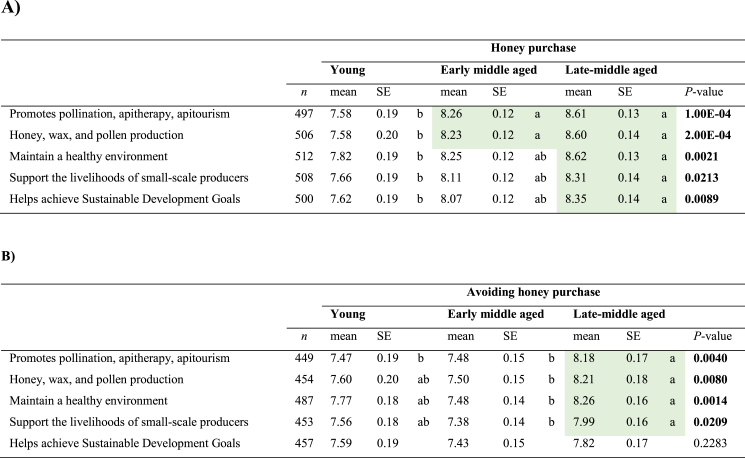
Age effects on interest in sustainable beekeeping management according to purchasing habits **A)** honey purchase and **B)** avoidance of honey purchase.

10 = Extremely interested; 9 = Very interested; 8 = Quite interested; 7 = Somewhat interested; 6 = Slightly interested; 5 = Neither interested nor uninterested; 4 = Slightly interested; 3 = Somewhat uninterested; 2 = Very uninterested; 1 = Extremely uninterested.

Abbreviation. **SE**= Standard error. Different letters indicate statistical differences related to age differences of different purchase habits using least significant difference test (*P* < 0.05). *P*-values were adjusted using Bonferroni’s method. Highlight in colour green are significantly higher mean values.

Regarding the honey purchase effect, early and late-middle-aged participants who purchase honey showed a higher level of interest across all sustainable items, except for the item “honey, wax, and pollen production” in the late-middle-aged group (*P* ≤ 0.05, Table 5). Remarkably, any of the sustainable benefits listed in this study did not lead to a significantly different level of interest among young participants who purchase or do not purchase honey (Table 5).

**Table 5:**
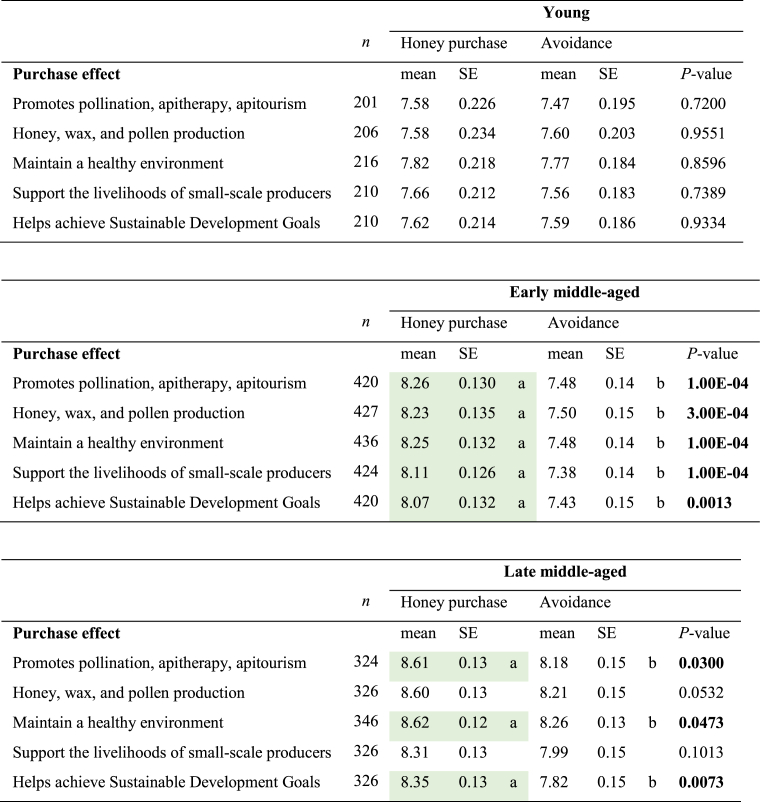
Honey purchasing habits effects on interest in sustainable beekeeping management according to age group.

10 = Extremely interested; 9 = Very interested; 8 = Quite interested; 7 = Somewhat interested; 6 = Slightly interested; 5 = Neither interested nor uninterested; 4 = Slightly interested; 3 = Somewhat uninterested; 2 = Very uninterested; 1 = Extremely uninterested.

Abbreviation. **SE**= Standard error. Different letters indicate statistical differences related to purchase habits differences of different age groups using least significant difference test (P < 0.05). P-values were adjusted using Bonferroni’s method. Highlight in colour green are significantly higher mean values.

### 3.5 Assessment of varying degrees of interest in the advantages of sustainable beekeeping management among habitual honey consumers

Since the GLMM results for the composite perceptions towards all sustainable management items showed a significant age and purchase effect, data was disaggregated by age. Late middle-aged participants reporting honey purchases showed statistically higher interest levels in the maintenance of a healthy environment if compared with the other benefits (*P* ≤ 0.05, Table 6). Interestingly, early middle-aged participants reported statistically higher interest levels of the promotion of pollination, apitherapy, apitourism, sustainable honey, wax, and pollen production and the maintenance of a healthy environment (*P* ≤ 0.05, Table 6). Surprisingly, no significant difference between the different benefits listed in this study of sustainable beekeeping management was reported by young participants.

**Table 6:**
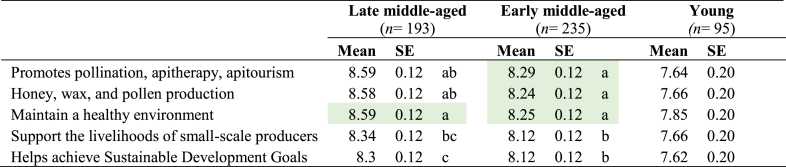
Age difference on interest of benefits of sustainable beekeeping management among participants who reported purchasing honey.

10 = Extremely interested; 9 = Very interested; 8 = Quite interested; 7 = Somewhat interested; 6 = Slightly interested; 5 = Neither interested nor uninterested; 4 = Slightly interested; 3 = Somewhat uninterested; 2 = Very uninterested; 1 = Extremely uninterested.

Abbreviation. **SE**= Standard error. Different letters indicate statistical differences related to age differences using least significant difference test (P < 0.05). P-values were adjusted using Bonferroni’s method. Highlight in colour green are significantly higher mean values.

## 4 Discussion

Honey consumption differed strongly across countries [69]. Nations such as Australia, which share a comparable estimated level of honey consumption with Belgium, have recently documented that the primary reasons for disliking honey are its characteristic flavour and texture [58]. These findings corroborate the results obtained in this study. Other aspects such as price and health problems, have been also cited as reasons for not consuming honey [70]. Furthermore, our results also indicate a clear age differentiation of negative associations towards consuming honey, where senior participants led with associations related to dislike and high cost and younger participants made associations with intolerance and dietary restrictions. Prior research notes that older consumers stated that convenience would not influence their food choices but previous knowledge about food and personal preferences was likely to be a factor when purchasing food [71]. Follow-up studies could expand on comparing the degree of the ethical, cultural, economic, and personal influence of disliking honey between different generations and geographical regions.

Existing research indicates that age, gender, level of education, income, and presence of children in the family positively influence honey consumption [29,72]. In addition, several studies revealed a growing interest in healthy food products that help, maintain or improve human health, well-being or reduce the risk of developing certain illnesses [73] In this context, there has been a growing surge of interest in honey and bee-derived products as a form of alternative and complementary medicine, particularly within the field of apitherapy, in recent years [74]. A recent study demonstrated that the therapeutic properties of honey play an important role in affecting consumers’ behaviour [72]. The conclusions drawn from examining the influence of socio-demographic factors on consumers' frequency of honey purchase, whether for dietary or medicinal purposes, and their reasons for avoiding honey consumption can serve as valuable insights for marketers and retailers. These insights can aid in the formulation and execution of effective marketing strategies and tactics.

Honey-purchasing decisions are influenced by health benefits, country of origin, producer reputation and ecological aspects [29,32,75]. According to Pocol et al.*,* (2022) the least important factors reported by Romanian consumers were discounts, promotion and brand followed by price and family budget [76]. Existing literature indicates that honey is purchased only a few times *per* year or monthly and mainly in hypermarkets supporting the results of this study [32,57,58]. According to our results and the literature, the frequency of honey purchase varies according to gender and age [55,57]. In this regard, recent studies indicate that men purchase and consume more honey than women, supporting the results of this study [55,77]. Although women are more involved in shopping, it appears that they have lower preferences for purchasing honey than men. Research shows a contradictory age effect on the purchase of organic food. In this regard, higher consumption, or intention to purchase organic food among older people has been reported [78,79], while the negative influence of age on the purchasing frequency of high-quality foods, including honey, has also been highlighted among young consumers [55,80]. This contradictory effect might be explained by the extrinsic factors influencing food choices such as personal preferences, level of knowledge, convenience, religion, belief, and intrinsic factors [71,75].

The food label is one of many sources of information consumers use to obtain knowledge about food items to make food purchasing decisions [81]. In the European context, health, environmental, or ethical claims are not mandatory for the food industry [82]. However, new laws on green claims are being adopted to tackle greenwashing and protect consumers and the environment [83]. Prior research notes that consumers seek more sustainable diets to maintain personal health and protect the environment [79,84]. During the COVID-19 pandemic, environmental concerns and health consciousness have become more important to consumers [85]. The results of this study also support the observation that, over the past few years, consumers have been seeking brands that offer sustainable production, help maintain a healthy environment, reduce the carbon footprint, and have ethical traits [84]. However, the ugly truth is that not all consumers are equally aware of the benefits of choosing green products, and overarching concerns with convenience shadow the healthy and environmental benefits [86].

Age is an important sociodemographic determinant of factors influencing sustainability awareness [87] and the results of this study suggest that older people are more interested in sustainable honey practices claims, denying the popular stereotype that the senior population is less likely to be environmentally responsible than the younger population also supported by existing literature [88]. Socio-demographic determinants are used as a basis for market segmentation and profiling environmentally-friendly consumers for developing sustainable marketing strategies and campaigns to reach and influence green purchases; thus it is imperative to align advertising content and format on consumer expectations for sustainable apiculture. Finally, policymakers in collaboration with local, regional, national, and international marketers could find means to promote the purchase of sustainable beekeeping management to specific consumers group, such as older individuals. The segment of elderly individuals is increasing in European countries and their food expenses are higher than other consumer segments. Therefore, stakeholders should adopt strategies to incentivize those consumers to buy sustainable beekeeping products by increasing their trust and increasing awareness of the impact on the planet. Implementing a certification system which guarantees both sustainable beekeeping management benefits and high-quality could constitute a further purchasing incentive for sustainable beekeeping products. Moreover, because conscious individuals are more prone to seeking out sustainable certification, policymakers can design more effective campaigns by using the right claim to advertise their public actions in favour of beekeeping and consequently the environment.

Although environmental claims have been studied extensively, ethical food labelling has been given little attention. Existing literature reported that consumers are shifting towards choosing or are willing to buy food products which support the livelihood of small-scale producers, social equity, and social justice; which avoid child labour; advocate ethical trait; ensure no discrimination; and promote traditional products [46,89]. It is important to highlight that - to the best of the authors’ knowledge -, there is no literature available regarding consumers’ interest in fairness and ethics in sustainable bee products. Conversely, sustainable beekeeping not only boosts the demand for novel products but also gives rise to fresh undertakings, such as apitherapy and api-tourism, which are closely linked to the preservation of cultural and historical legacies [70,90]. Beekeepers should promote the benefits of consuming bee products that follows sustainable beekeeping management as a sign of not only quality but ethical, environmental, cultural, and global benefits which should represent an important element for producers and distributors adding value to their competitive bee products market.

This study has strengths and weakness. Motives for and against participation could be possible starting points for approaches to overcome recruitment difficulties. The response of a total of 1100 consumers from one country is relatively low and provides only a small representation of the European context. However, our findings represent the voice of key stakeholders influencing honey imports and describe the reality of one of the leading members of the European Union. In addition, the methodology used of this study has potential limitations, such as the potential confounding variables that could influence the results of the present study include factors such as educational level, occupation, and country of origin of respondents may also play a role. They are therefore subject to bias. To address this limitation, the use of methodological strategies such as wider geographic reach, high response rates, low straight lining, and strengthen the validity of the results.

On the other hand, our inclusion criteria can be considered a strength. The survey was conducted online, allowing participants to respond at their own pace and in a private setting to encourage honest responses. Importantly, there are no similar studies conducted in other European countries targeting consumers with low honey consumption. In fact, most research evaluates the perception of consumers for specific claims, such as health, nutritional and social sustainability individually [91,92], while this study aimed to understand consumers’ views on vary target environmental claims to understand the market dynamics of sustainable apiculture.

Despite the great importance of the environmental, ethical, social, and global benefits associated with sustainable beekeeping management, consumers are often unaware of beekeeping practices. Existing literature provides evidence that consumers correlate the level of industrial processing with the perceived healthiness [93]. With this regard, great efforts have been made to promote awareness of the impact of the whole food supply chain - including the farming, processing, transportation, and consumption process - using sustainable claims as a marketing tool which influences consumer needs and beliefs [83]. Previous research shows that consumers care about environmentally sustainable attributes or on-farm practices along the food supply chain, but they lack knowledge of definitions and how to process the information [39]. Buying labelled products allows consumers to take part in making informed decisions and raising their voices about issues related to the ethics and food production process [94]. This study provides a framework of consumers interest on the benefits of sustainable beekeeping management in Belgium from different perspectives. Consumers' purchasing habits are evolving due to shifting perceptions concerning ethics, culture, the environment, food production, and global factors. Hence, it is imperative for managers, practitioners, stakeholders, and policymakers to take these developments into account in order to align claims with emerging consumer concerns and requirements, thereby bolstering support for sustainable beekeeping practices.

## 5 Conclusion

According to our survey findings, Belgian consumers who regularly purchase honey tend to exhibit a higher level of interest in the environmental advantages associated with sustainable beekeeping management, in comparison to cultural, ethical, production of bee products, or global benefits. The act of avoiding honey resulted in lower perception scores, potentially due to a greater degree of scepticism towards the advantages of sustainable beekeeping practices when compared to those who purchase honey. We suggest that improving communication might increase the purchase of sustainable honey. The hybrid method employed in this study yielded valuable insights by combining the analysis of multiple-choice responses through CA with the examination of survey questions using GLMM analysis. The constraints of this study encompass the possibility that when consumers are asked about their level of interest in the benefits of sustainable beekeeping management, they might be inclined to report a heightened level of interest. Furthermore, terms like 'apitherapy,' 'apitourism,' and 'SDGs' may not be direct and could be unfamiliar to many, whereas terms like 'pollination services' and 'honey production' are more straightforward and widely recognized. This familiarity with the terms could have played a role in the observed effects in this study, especially in the context of comprehending cultural and ethical implications. This study presented information about global benefits when sustainable beekeeping management is used. Comparison of global and personal benefits could be investigated in further research, as well as, how these perceptions could vary between generations and larger age groups from different geographical regions. Concerning the methodology adopted in this study, the use of multiple answers led to a reduced response rate, dependability, and reproducibility, while the use of validated survey scales led to reliable results due to the use of statistical analyses and interpretations of the test results. To a certain extent, the use of open-ended and validated survey scales led to conclusions with a reduced risk for application and future studies. Nonetheless, further research is required to expand upon the scant body of literature regarding consumers' perceptions of sustainable beekeeping management. This is essential to validate the findings and explore their broader applications in consumer and marketing research, as well as in the realm of sustainable labelling.

As the emphasis on enhancing bee health through environmental improvements grows, future studies should strive to obtain results that offer insights closer to predicting real-world behaviour. This would help uncover potential obstacles to selecting sustainable honey and drive positive changes in agriculture, beekeeping practices, and honey demand. Redirecting public attention and policy from conventional beekeeping management to evidence-based conservation for bees and non-bee species is critical to increase the resilience of agricultural and natural ecosystems and inform changes in the standard for organic certifications. Organic honey and bee products are practical examples that are generated through sustainable foods policies.

## CRediT authorship contribution statement

**Jatziri Mota-Gutierrez:** Writing – review & editing, Visualization, Methodology, Investigation, Data curation. **Stefano Massaglia:** Writing – review & editing, Resources, Project administration, Funding acquisition. **Valentina Maria Merlino:** Writing – review & editing, Supervision. **Federica Rosa:** Project administration, Investigation, Funding acquisition. **Andrea Viberti:** Project administration, Investigation, Funding acquisition. **Simone Blanc:** Writing – review & editing, Validation, Supervision, Methodology, Conceptualization.

## Data availability statement

The data will be available upon reasonable request through corresponding authors.

## Funding sources

This research did not receive any specific grant from funding agencies in the public, commercial, or not-for-profit sectors.

## Declaration of competing interest

The authors declare that they have no known competing financial interests or personal relationships that could have appeared to influence the work reported in this paper.
