# Supplementary material for: Level of interest among Belgian consumers of the cultural, environmental, ethical, and global benefits of sustainable beekeeping management
Source: Heliyon. 2024 Dec 9;10(24):e40928. doi: 10.1016/j.heliyon.2024.e40928 (PMC11699047; doi:10.1016/j.heliyon.2024.e40928)
Supplement: Multimedia component 1 [file mmc1.docx]

**Supplementary Table**

**Table S1.** Questionnaire

Section 1. **Honey purchase habits**

1. Do you consume honey?

Please Choose ONE of the options below

- Yes
- No

1. If not…Why you do not consume honey?

Please Choose as many options as possible below

- Health problems
- Intolerance
- Religion
- Not high-quality product
- Dislike
- Untrust
- High cost

1. If yes, how often do you purchase honey?

Please Choose ONE of the options below

- More than 5 times a week
- 3 - 5 times per week
- 1 - 3 times per week
- Once a month
- Once *per* year
- Special occasions
- Never

1. Where do you usually purchase honey?

Please Choose ONE of the options below

- Large supermarkets
- Specialized stores
- Restaurants
- On-line
- None

Section 2. **Sustainable beekeeping practice**

How would you rate your interest towards the following statements? Sustainable beekeeping managements…*Mandatory for all participants

Please answer the following statements by circling ONE number beside each statement in an appropriate column. Circle any number to describe your personal degree of agreement. Make your ratings promptly, based on your first impression. No right or wrong responses exist, only your personal opinion counts.

|  | 1 | 2 | 3 | 4 | 5 | 6 | 7 | 8 | 9 | 10 |
| --- | --- | --- | --- | --- | --- | --- | --- | --- | --- | --- |
|  | Not important at all | Very uninterest | Somewhat uninterest | Slightly uninterest | Neither interest or uninterest | Slightly interest | Somewhat interest | Quite interest | Very interest | Extremely interest |
| promotes pollination, apitherapy and api-tourism. |  |  |  |  |  |  |  |  |  |  |
| produces honey, wax, propolis and pollen. |  |  |  |  |  |  |  |  |  |  |
| helps maintain a healthy environment. |  |  |  |  |  |  |  |  |  |  |
| supports the livelihoods of small-scale producers. |  |  |  |  |  |  |  |  |  |  |
| helps achieve the Sustainable Development Goals. |  |  |  |  |  |  |  |  |  |  |

Section 3. **Demographic Information**

1. Sex

Please Choose ONE of the options below

- Female
- Male
- Don’t respond

11) How old are you?

12) What is your current occupation?

Please Choose ONE of the options below

- Student
- Independent worker
- Private-sector worker
- Public-sector worker
- Retired
- Unemployed
- Not seeking work
- Other work

13) What is your household size?

Please Choose ONE of the options below

- 1 component
- 2 components
- 3 components
- 4 components
- 5 or More components

14) What is your annual family income?

Please Choose ONE of the options below

- Less than 10 000 €
- Between 10 000 and 15 000 €
- Between 15 000 and 20 000 €
- Between 20 000 and 25 000 €
- Between 25 000 and 30 000 €
- Between 30 000 and 40 000 €
- Between 40 000 and 60 000 €
- Between 60 000 and 80 000 €
- More than 80 000 €
- Not respond.
